# MEG3, HCN3 and linc01105 influence the proliferation and apoptosis of neuroblastoma cells via the HIF-1α and p53 pathways

**DOI:** 10.1038/srep36268

**Published:** 2016-11-08

**Authors:** Weitao Tang, Kuiran Dong, Kai Li, Rui Dong, Shan Zheng

**Affiliations:** 1 Department of Pediatric Surgery, Children’s Hospital of Fudan University, Shanghai, China

## Abstract

The purpose of this study was to investigate the differential expression and functional roles of long non-coding RNAs (lncRNAs) in neuroblastoma tissue. LncRNA microarrays were used to identify differentially expressed lncRNAs between tumor and para-tumor tissues. In total, in tumor tissues, 3,098 and 1,704 lncRNAs were upregulated and downregulated, respectively. *HCN3* and *linc01105* exhibited the higher expression (P < 0.05; P < 0.01, respectively) in neuroblastoma tissue, whereas *MEG3* displayed the lower expression (P < 0.01). *HIF-1α* expression was negatively correlated with cell proliferation in the *linc01105* KD group. In addition, *Noxa* and *Bid* expression was positively correlated with cell apoptosis. Moreover, *linc01105* knockdown promoted cell proliferation, whereas *MEG3* overexpression inhibited proliferation. Finally, *linc01105* knockdown, *MEG3* overexpression and *HCN3* knockdown all increased apoptosis. The correlation coefficients between those three lncRNAs and the International Neuroblastoma Staging System (INSS) stage were −0.48, −0.58 and −0.55, respectively. In conclusion, we have identified lncRNAs that are differentially expressed in neuroblastoma tissues. The lncRNAs *HCN3, linc01105*, and *MEG3* may be important in biological behaviors of neuroblastoma through mechanisms involving p53 pathway members such as *HIF-1α, Noxa*, and *Bid*. The expressions of *MEG3, HCN3* and *linc01105* are all negatively correlated with the INSS stage.

Neuroblastoma is the most common type of extracranial solid tumor in children; it arises from the neural crest of the sympathetic nervous system and typically occurs in the adrenal medulla^1^. Neuroblastoma may regress spontaneously or differentiate into a ganglioneuroblastoma or benign ganglioneuroma, particularly in infants. The prognosis is good in children diagnosed with neuroblastoma at 1 year of age; however, undifferentiated neuroblastomas diagnosed after 5 years of age have a poor prognosis^2^,^3^.

In addition to protein-coding mRNAs, a large number of non-coding RNAs have been identified with structural, regulatory, and unknown functions. Long non-coding (lnc)RNAs, which are >200 nt in length^4^,^5^, have been implicated in gene regulation at the transcriptional and post-transcriptional levels^6^. The dysregulation of lncRNAs is a primary feature of human cancers, including prostate, breast, colon, and gastric cancers^7^,^8^,^9^,^10^. LncRNAs are also aberrantly expressed in disorders such as Beckwith-Wiedemann, DiGeorge, and Down’s syndromes^11^,^12^,^13^,^14^,^15^,^16^. However, the role of lncRNAs in neuroblastoma has not been fully investigated. Here, we have investigated the role of lncRNAs in the pathogenesis of neuroblastoma.

## Results

### LncRNA and mRNA expression profiles

After quantile normalization of the raw data, the expression profiles of 36,178 lncRNAs and 24,992 mRNAs were obtained for tumor and para-tumor tissues (Supplementary Fig. 1). The log2 distributions of the ratios of lncRNAs to mRNAs were similar for the two groups.

### lncRNAs and mRNAs are differentially expressed in neuroblastoma tissues and para-tumor tissues

To identify differentially expressed lncRNAs and mRNAs between tumor and para-tumor tissues, we performed fold-change filtering (values ≥ 2). We found that 3,098 lncRNAs and 2,526 mRNAs were upregulated, whereas 1,704 lncRNAs and 2,604 mRNAs were downregulated, in neuroblastoma tissues compared to control samples (Supplementary Fig. 2).

### Pathway analysis

The pathway analysis revealed 44 downregulated and 59 upregulated pathways in neuroblastoma tissue compared to control tissue. The p53 signaling pathway, which is associated with tumorigenesis, was among the upregulated pathways (enrichment score = 1.44; P = 0.036).

### CNC network analysis

The upregulated p53 signaling pathway genes included BH3-interacting domain death agonist (*BID*), phorbol-12-myristate-13-acetate-induced protein (*PMAIP*), mitochondrial ribonuclease P protein 2 precursor 2, sestrin 3, cyclin (*CCN*) D1, *CCNB1*, and *CCNE2*. The CNC analysis revealed that the following lncRNA genes were differentially expressed between tumor and para-tumor tissues: maternally expressed gene (*MEG*) 3, hyperpolarization-activated cyclic nucleotide (*HCN*) 3, and long intergenic non-protein-coding RNA (*linc*) 01105 (P < 0.001). Pearson correlation coefficients were calculated as follows: *lncRNA-MEG3* and *PMAIP1* (−0.989), *lncRNA-HCN3* and *BID* (0.988) and *linc01105* and *BID* (0.987) (Supplementary Fig. 3).

#### Validation of differential lncRNA expression by qRT-PCR

Consistent with the microarray results, the qRT-PCR analysis revealed that the expression levels of *HCN3* (P < 0.05) and *linc01105* (P < 0.01) were higher, whereas *MEG3* expression was lower (P < 0.01), in neuroblastoma tissue compared to para-tumor tissue (Supplementary Fig. 4).

### Cell proliferation (CCK8 test) results

**Figure 1:**
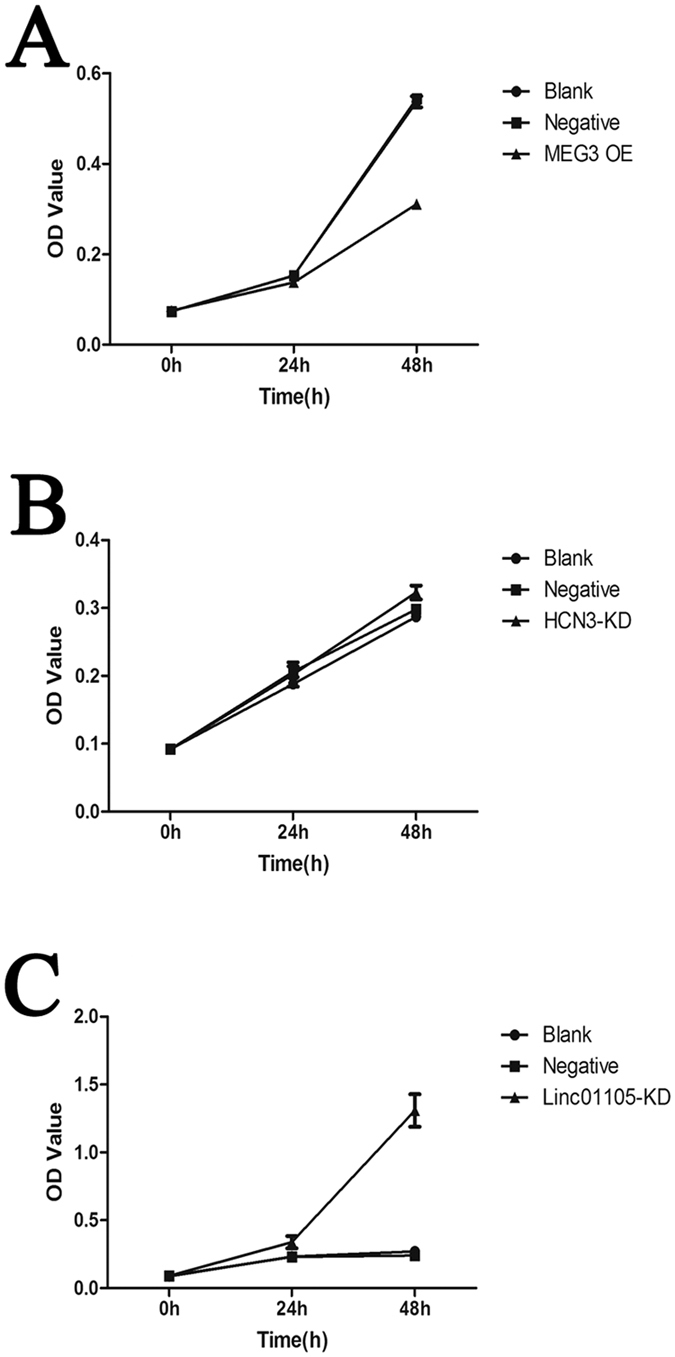
Effect of lncRNA expression on cell proliferation at different time points. (**A**) Between 0 and 24 h and from 24 to 48 h, cell proliferation decreased in the *MEG3* OE group compared to the blank group (P = 0.002; P < 0.001). (**B**) Between 0 and 24 h and from 24 to 48 h, cell proliferation increased in the *linc01105* KD group compared to the blank group (P = 0.022; P < 0.001). (**C**) Between 0 and 24 h and from 24 to 48 h, cell proliferation was similar in the *HCN3* KD and blank groups (P = 0.269; P = 0.095).

The change between the optical density (OD) values from 0 to 24 h was 0.064 ± 0.002 in the *MEG3* overexpression (OE) group and 0.124 ± 0.01 in the *linc01105* knockdown (KD) group. The *MEG3* OE group and the *linc01105* KD group were significantly different from their blank groups (*MEG3* OE, P = 0.002; *linc01105* KD P = 0.022) The change between the OD values from 24 to 48 h was 0.173 ± 0.011 in the *MEG3* OE group and 0.97 ± 0.076 01 in the *linc01105* KD group. Both groups were significantly different from their controls (P < 0.001) There were no differences between the *HCN3* KD group and the controls at any time point (0 to 24 h, P = 0.269; 24 to 48 h, P = 0.095) (Fig. 1).

### Apoptosis results

**Figure 2:**
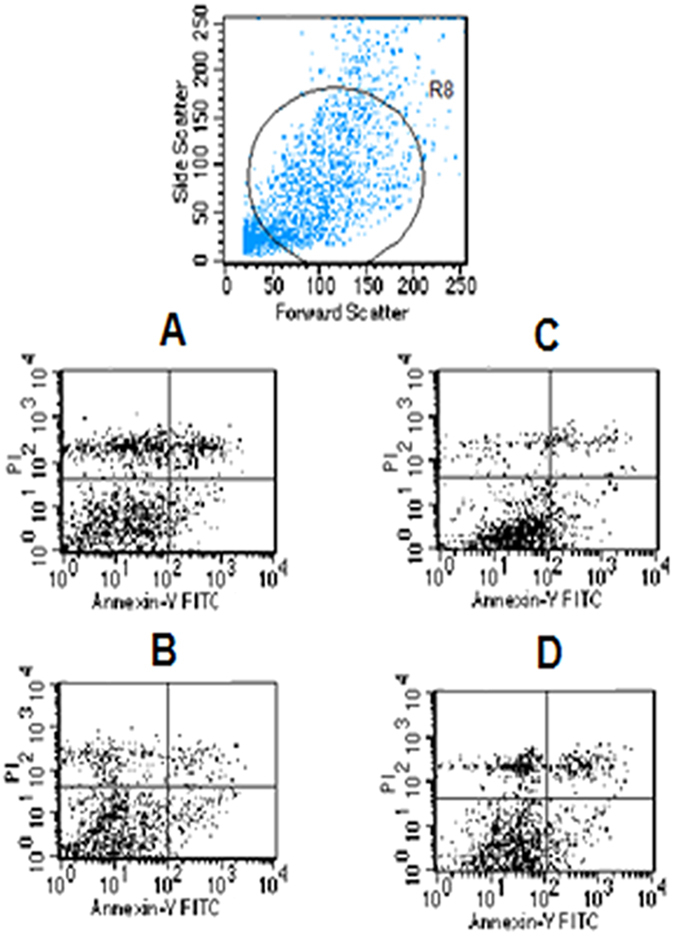
Effect of lncRNA expression on apoptosis. (**A**) Blank group. (**B**) *MEG3* OE group. (**C**) *HCN3* KD group. (**D**) *linc01105* KD group.

The numbers of apoptotic cells were as follows: blank group, 398 (6.7%); *MEG3* OE group, 394 (8.7%); *HCN3* KD group, 600 (11.4%); and *linc01105* KD group, 534 (9.7%). All OE and KD groups had significantly higher levels of apoptosis than the blank group (P < 0.001) (Fig. 2).

### mRNA expression of genes of interest

**Figure 3:**
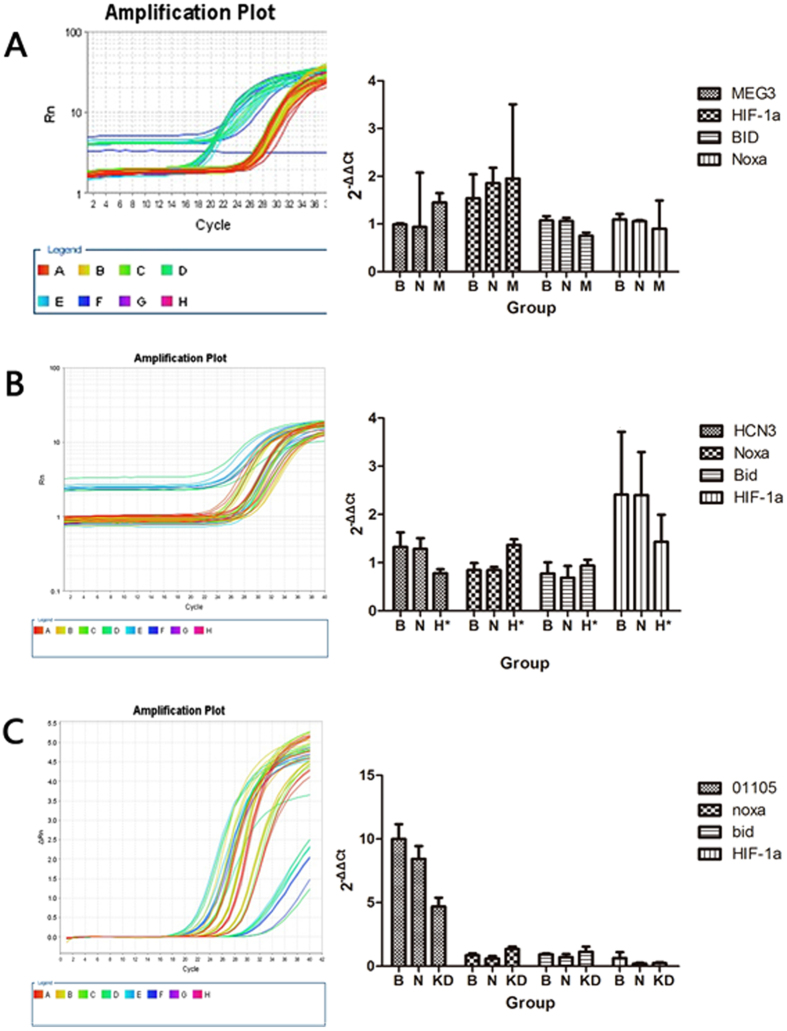
The conditions of target genes and relevant genes in each group. (**A**) Amplification plots of *lncRNA-MEG3, HIF-1α, Noxa* and *Bid* mRNA expression in the blank, negative control and *MEG3* OE groups. (**B**) Amplification plots of *lncRNA-HCN3, HIF-1α, Noxa* and *Bid* mRNA expression in the blank, negative control and *HCN3* KD groups. (**C**) Amplification plots of *lncRNA-linc01105, HIF-1α, Noxa* and *Bid* mRNA expression in the blank, negative control and *linc01105* KD groups. B, Blank group; N, Negative control group; H, *HCN3* KD group; M, *MEG3* OE group; KD, 01105 KD group.

The relative expression (2^−ΔΔCt^) of *Bid* mRNA in the *MEG3* OE group was 0.751 ± 0.071, which was lower than the blank group (P = 0.009). The relative expression (2^−ΔΔCt^) of *Noxa* mRNA was 1.368 ± 0.118 in the *HCN3* KD group and 1.354 ± 0.19 in the *linc01105* KD group. The expression in both groups was higher than in the controls (P = 0.009; P = 0.025). In the *MEG3* OE group, the relative expression of *HIF-1α* (P = 0.683) and *Noxa* mRNA (P = 0.606) was similar to the blank groups. In the *HCN3* KD and *linc01105* KD groups, the relative expression of *HIF-1α* and *Bid* mRNA was similar to the controls (Fig. 3).

### Expression of relevant proteins

**Figure 4:**
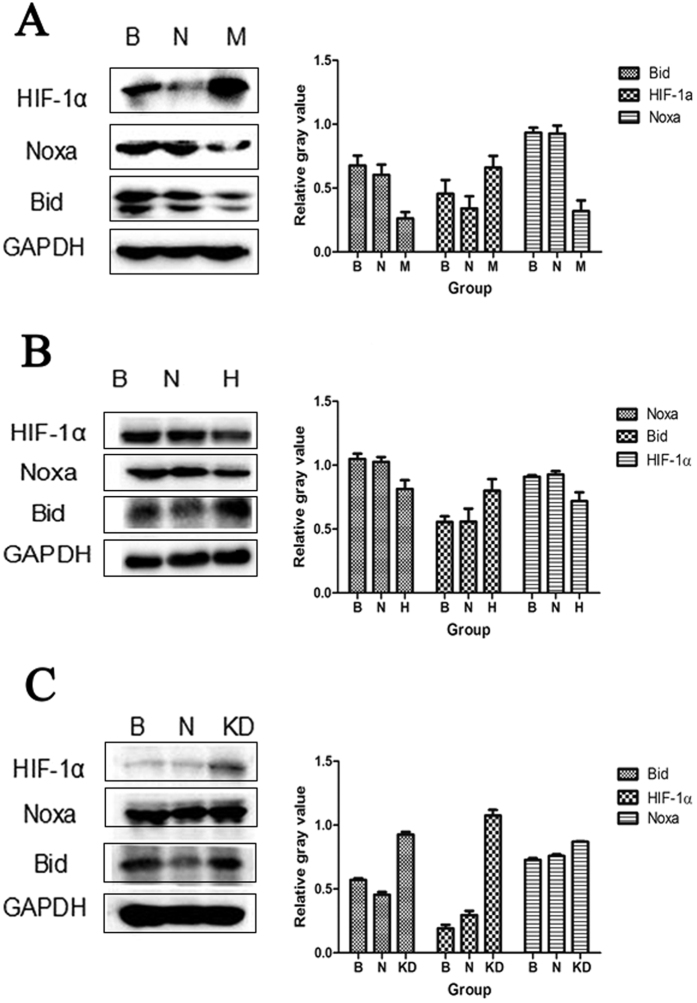
The effect of lncRNAs on HIF-1α, Bid and Noxa protein expression. (**A**) Expression of *HIF-1α, Noxa* and *Bid* in the blank, negative control and *MEG3* OE groups. (**B**) Expression of *HIF-1α, Noxa* and *Bid* in blank, negative control and *HCN3* KD groups. (**C**) Expression of *HIF-1α, Noxa* and *Bid* in blank, negative control and *linc01105* KD groups. B, Blank group; N, Negative control group; H, *HCN3* KD group; M, *MEG3* OE group; KD, *linc01105* KD group. Full-length blots were shown as supplementary figure 6 in supplementary information.

The effects of *MEG3, HCN3* and *linc01105* OE or KD on the protein expressions of HIF-1α, Noxa and Bid were analyzed. The expression of Noxa protein was 0.319 ± 0.084 in the *MEG3* OE group and 0.815 ± 0.068 in the *HCN3* KD group, both of which were lower than the blank groups (P < 0.001; P = 0.007). However, the expression of Noxa increased in the *linc01105* KD group compared to the blank group (0.871 ± 0.003; P = 0.002). In addition, the expression of Bid protein was 0.8 ± 0.092 in the *HCN3* KD group and 0.927 ± 0.021 in the *linc01105* KD group, both of which were higher than Bid expression in the blank groups (P = 0.014; P < 0.001). However, Bid expression was reduced in the *MEG3* OE group compared to the blank group (0.262 ± 0.05; P = 0.001). The expression of HIF-1α protein decreased in the *HCN3* KD group (0.719 ± 0.069, P = 0.009) and increased in the *linc01105* KD group (1.075 ± 0.046, P < 0.001). HIF-1α expression was not affected by *MEG3* OE (0.661 ± 0.091; P = 0.065) (Fig. 4).

### Immunofluorescence results

**Figure 5:**
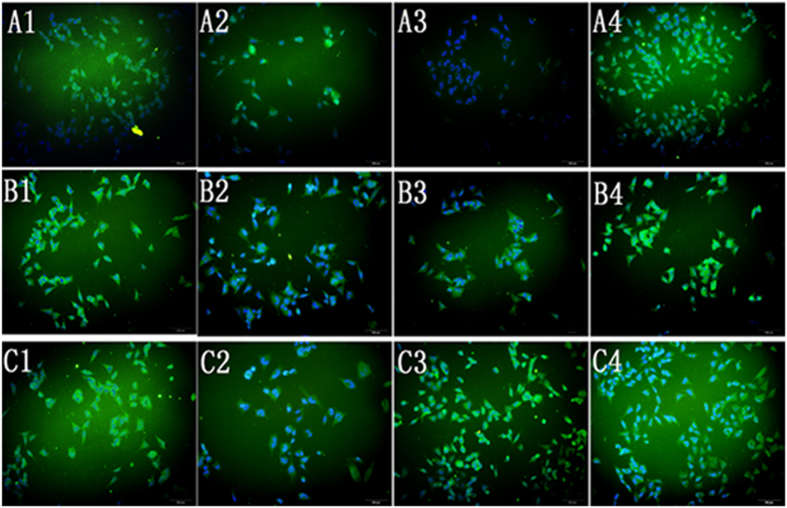
Immunofluorescence provides information about protein expression and localization. (A1–A4) Expression of *HIF-1α* protein in the blank, *MEG3* OE, *HCN3* KD and *linc01105* KD groups. (B1–B4) Expression of *Noxa* protein in the blank, *MEG3* OE, *HCN3* KD and *linc01105* KD groups. (C1–C4) Expression of *Bid* protein in the blank, *MEG3* OE, *HCN3* KD and *linc01105* KD groups.

HIF-1α protein was highly expressed in the *linc01105* KD group, weakly expressed in the *HCN* KD group and did not change expression in the *MEG3* OE group. Positive HIF-1α staining was observed in the cytoplasm and nucleus of the cells in the blank, *MEG3* OE and *linc01105* KD groups, but its expression was restricted to the cytoplasm in the *HCN3* KD group. Noxa staining was strong in the *linc01105* KD group but weak in the *MEG3* OE and *HCN3* KD groups, and staining was observed in the cytoplasm and nucleus of the cells in all groups. Bid protein was strongly expressed in *HCN* KD and *linc01105* KD cells but was weakly expressed in the cells overexpressing *MEG3*. Bid staining was observed in the cytoplasm and nucleus of the cells in all groups (Fig. 5).

### Correlation between lncRNA expression and neuroblastoma International Neuroblastoma Staging System (INSS) stage

The expression of the three lncRNAs (*MEG3, HCN3, and linc01105*) was negatively correlated with INSS stage to a moderate extent. The Spearman correlation coefficient values were: *MEG3*, −0.48; *HCN3*, −0.58; and *linc01105*, −0.55.

## Discussion

Neuroblastoma is the most common type of solid tumor in infants, accounting for 8–10% of all childhood cancers and approximately 15% of all cancer-related deaths in children^1^,^17^. The etiology of neuroblastoma is heterogeneous and includes amplification of the *MYCN* or anaplastic lymphoma kinase oncogenes and the allelic loss of chromosomes 1p, 3p, or 11q. These etiologies are associated with varying clinical outcomes^18^ and affect chromosomes or protein-coding genes. The dysregulation of lncRNA expression has been linked to various diseases^19^,^20^,^21^,^22^,^23^. However, studies of the roles of lncRNAs in neuroblastoma are lacking. In the present study, we identified 4,802 lncRNAs and 5,130 mRNAs that were differentially expressed in neuroblastoma tissues. ENST00000523785, ENST00000393515, uc010fhf.3, ENST00000513011, and uc003wtj.3 were the most significantly upregulated lncRNAs, whereas NR_051975, ENST00000570869, ENST00000555539, uc001lva.4, and ENST00000570945 were the most significantly downregulated.

The p53 signaling pathway has been implicated in the development of various carcinomas, and recent studies suggest that lncRNAs regulate gene expression in some tumors by modulating p53 pathway members. For example, metastasis-associated lung adenocarcinoma transcript 1 has been linked to lung, breast, colon and liver cancer^24^,^25^,^26^,^27^,^28^. Here, a CNC network analysis implicated the lncRNAs *HCN3, linc01105*, and *MEG3* in p53 signaling. *HCN3* encodes a type of pacemaker channel in spontaneously contractile cells and plays an important role in regulating cell excitability^29^,^30^. *MEG3* is a maternally expressed imprinted gene that inhibits the growth of ectopic human cancer cells and is considered to be a tumor suppressor^31^. For example, the downregulation of MEG3 was found to be associated with poor overall survival in osteosarcoma^32^. *linc01105* is an RNA gene of undetermined function. Hypoxia is considered to be an inducer of p53 signaling^33^, and evidence suggests that increased p53 expression under hypoxic conditions is dependent upon HIF-1α^34^,^35^. In this study, we examined whether these three lncRNAs were involved in the pathogenesis of neuroblastoma and whether the underlying mechanisms involved the p53 and HIF-1α pathway.

We identified *MEG3, HCN3* and *linc01105* as lncRNAs that are relevant to neuroblastoma. *MEG3* is expressed in many normal tissues, but its expression is lost in many primary human tumors^36^. *HCN3* represents one of four HCN channel isoforms (HCN1-HCN4)^37^. Little is known about the role of *linc01105*. In this study, the overexpression of *MEG3* inhibited the proliferation of BE(2)-C neuroblastoma cells but promoted their apoptosis. Knockdown of HCN3 had no effect on cell proliferation but promoted cell apoptosis. Knockdown of *linc01105* expression promoted BE(2)-C cell proliferation and apoptosis. Altered lncRNA expression influenced the levels of HIF-1α mRNA and protein in neuroblastoma cells. MEG3 OE had no effect on the levels of HIF-1α mRNA or protein. Knockdown of HCN3 and linc01105 had no effect on the levels of HIF-1α mRNA. HCN3 KD resulted in the downregulation of HIF-1α protein expression, but linc01105 KD led to elevated HIF-1α protein expression. This result suggests that MEG3, HCN3 and linc01105 regulate the expression of HIF-1α by post-transcriptional mechanisms. Evidence suggests that HIF-1α is expressed in several tumor types as a result of hypoxia and the expression of certain oncogenes^38^,^39^, and it is involved in cell proliferation^40^,^41^. Our study suggests that linc01105 influences proliferation by modulating HIF-1α protein expression. However, it is unknown whether MEG3 and HCN3 control cell proliferation through the regulation of HIF-1α expression.

Noxa is a member of the BH3-only subset of the pro-apoptotic BCL-2 protein family. BCL-2 proteins are important regulators of the intrinsic apoptotic pathway^42^,^43^. BH3-interacting death domain agonist (BID) also belongs to the BH3-only subset of BCL-2 proteins, and it plays a pro-apoptotic role in cell stress responses^44^,^45^,^46^,^47^,^48^,^49^. KD of *HCN3* increased Bid expression and levels of cell apoptosis. In addition, *linc01105* KD increased the protein-level expression of Noxa and Bid and enhanced cell apoptosis. Noxa and Bid are both apoptosis-associated proteins in the p53 pathway, and our findings suggest that *HCN3* and *linc01105* regulate apoptosis via the Noxa and Bid proteins. *MEG3* OE reduced the expression of Noxa and Bid but increased the levels of apoptosis. The abnormal expression of *MEG3* was shown to decrease cell growth and promote apoptosis^50^; therefore, *MEG3* may directly promote apoptosis in neuroblastoma cells. Our study also revealed the relationship between Bid and Noxa expression and cell proliferation. Reduced expressions of Bid and Noxa inhibited cell proliferation, whereas increased and decreased Bid and Noxa expressions, respectively, had no effect on cell proliferation. However, given that the increases in Bid and Noxa expressions promoted cell proliferation, it is possible that Bid and Noxa influence cell proliferation cooperatively.

The expressions of *MEG3, HCN3* and *linc01105* were all correlated negatively with the INSS neuroblastoma stage; low and high expressions of these genes were associated with early and late tumor stages, respectively. These correlations were consistent with the regulatory roles of these lncRNAs in neuroblastoma cells. These findings suggest that *MEG3, HCN3* and *linc01105* may predict the prognosis of neuroblastoma.

In summary, this study has identified lncRNAs and mRNAs that are differentially expressed in neuroblastoma and that may serve as biomarkers for prognosis or disease progression. *HCN3, linc01105*, and *MEG3* are likely to be important in the biological behaviors and progression (stage) of neuroblastoma and may act via a mechanism involving HIF-1α, Noxa and Bid, and thus the p53 pathway.

## Materials and Methods

### Clinical samples

Tumor and para-tumor tissue samples (n = 6) were collected from patients who underwent surgery between December 2011 and December 2013; these samples were and stored at −80 °C. Tumor tissue specimens were confirmed as neuroblastomas by a pathologist. Para-tumor tissue samples consisted of adrenal gland tissue, and the absence of tumor cells was confirmed by microscopy. The age of the patients from whom the tissue samples were collected were 2 months, 10 months, 7 years, 3 years, 6 years and 3 months, respectively. We confirm that all of the methods were performed in accordance with relevant guidelines, and all of the experimental protocols were approved by the Ethics Committee of the Children’s Hospital of Fudan University. We also confirm that informed consent was obtained from the parents or legal guardians of the subjects.

### RNA isolation

The tissue samples were washed three times with cold phosphate-buffered saline and dissolved in TRIzol reagent (Invitrogen, USA). Total RNA was isolated using the RNeasy Mini kit (Qiagen, Germany) according to the manufacturer’s protocol, which included DNase digestion. The purity and concentration of the RNA samples were determined by measuring absorbance with an ND-10009 NanoDrop spectrophotometer (Thermo Fisher Scientific, USA). Samples with an OD 260/280 nm close to 2.0 and an OD 260/230 nm >1.8 were deemed sufficiently pure for use in the experiments.

### RNA labeling and array hybridization

To identify differentially expressed mRNAs, RNA samples were amplified and labeled using the Quick Amp Labeling kit (Agilent Technologies, USA) and hybridized onto whole genome microarrays (Agilent Gene Expression Hybridization kit). The Human LncRNA Microarray v3.0 platform (Agilent Technologies) was used to identify differentially expressed lncRNAs. After hybridization and washing, the arrays were scanned (G2565BA; Agilent Technologies) according to the manufacturer’s recommendations.

### Data analysis

Agilent Feature Extraction software was used to analyze the acquired images. Quantile normalization and raw data processing were conducted using GeneSpring GX V12.0 software (Agilent Technologies). LncRNAs and mRNAs with “present” or “marginal” (“all targets value”) flags were selected for further analysis. The differential expression of lncRNAs and mRNAs between tumor and para-tumor samples was determined by fold-change (>2-fold) filtering.

### LncRNA classification and pathway analysis

Functional analyses were performed to identify to which Kyoto Encyclopedia of Genes and Genomes (KEGG) pathways the differentially expressed genes belonged. Pathways with a P-value of ≤ 0.05 were considered significant.

### Coding/non-coding gene co-expression (CNC) network analysis

A subset of the data was selected according to the coding list. The Pearson correlation coefficients between coding and non-coding RNAs were determined, with an r-value of ≥0.985 indicating a relationship. A CNC network was generated based on the results using Cytoscape (v2.8.1).

### Evaluation of lncRNA expression by quantitative real-time (qRT)-PCR

The expression of lncRNAs identified by microarray analysis was confirmed by qRT-PCR. cDNA was synthesized from total RNA using the PrimeScript reverse transcription reagent kit with a genomic DNA Eraser (Takara, Japan). Primers were designed to amplify the three lncRNAs, and amplification was performed using a LightCycler 480 instrument (Roche Applied Science, Switzerland). The 10-μl PCR reactions included 1 μl of cDNA template and 5 μl of SYBR Premix Ex Taq II (Takara). The reaction conditions were as follows: 95 °C for 1 min, followed by 40 cycles of 95 °C for 5 s, 60 °C for 10 s, and 72 °C for 15 s. The reactions were run in triplicate. Threshold cycle (Ct) values were determined using default threshold settings, and the mean Ct was determined from duplicate PCR reactions. LncRNA expression levels were measured and normalized to the expression of human *18s* rRNA using the 2^−ΔΔCt^ method.

### Functional analyses of target genes

#### Gene overexpression and knockdown

Lentiviral overexpression and knockdown vectors were constructed for lncRNAs of interest. The BE(2)-C neuroblastoma cell line was purchased from the American Type Culture Collection (ATCC) and cultured in complete Dulbecco’s modified Eagle medium (DMEM) (Gibical, Australia) supplemented with 10% fetal bovine serum (Gibical) in a 37 °C incubator maintained at 5% CO_2_. BE(2)-C cells were infected with 100 μl of lentiviral vectors in 900 μl of DMEM plus 5 μg/ml polybrene. The cells were incubated with viruses for 12 h at 37 °C and 5% CO_2_.

#### Cell proliferation

Three groups were tested using the CCK8 kit (Dojindo, Japan): (1) the overexpression or knockdown group, (2) the negative control group and (3) the blank group. BE(2)-C neuroblastoma cells were seeded in 96-well plates (3,000 cells/well), and the OD values of cells in the three groups were measured at two different time points (24 and 48 h).

#### Apoptosis detection

The target genes were either overexpressed (OE) or knocked down (KD) in BE(2)-C cells, and apoptosis levels relative to the control cells were quantified using the Annexin V-FITC/PI Apoptosis Detection kit (Dojindo, Japan). Apoptosis was analyzed using a FACScan flow cytometer.

#### RNA extraction and qRT-PCR

RNA was extracted from BE(2)-C cells using the RNeasy kit (Omega, USA). cDNA was produced using the PrimeScript Reverse Transcriptase reagent kit with genomic DNA Eraser (Perfect Real-Time) (Takara). qRT-PCR was performed using an Applied Biosystems 7900 HT qPCR machine and SYBR Premix Ex Taq (Tli RNaseH Plus) (Takara). The relative abundance of cDNA was calculated using the relative standard curve method.

#### Western blot analysis

The cells were lysed in radio immunoprecipitation assay (RIPA) buffer for 30 min, and the proteins were separated by sodium dodecyl sulfate-polyacrylamide gel electrophoresis (SDS-PAGE) and transferred to polyvinylidene fluoride (PVDF) membranes. The membranes were then blotted with rabbit anti-HIF-1α (Abcam, 1:1000), rabbit anti-noxa (Abcam, 1:1000), rabbit anti-bid (Abcam, 1:500) or mouse monoclonal anti-GAPDH (Abcam, 1:8000) primary antibodies in 5% non-fat dry milk, followed by HRP-conjugated anti-rabbit (Abcam, 1:1000) or anti-mouse secondary antibodies (Abcam, 1:1000).

#### Immunofluorescence

BE(2)-C cells were seeded in 48-well plates and labeled with the following primary antibodies: anti-HIF-1α (Abcam, 1:500), anti-noxa (Abcam, 1:500) and anti-bid (Abcam, 1:500). Primary antibodies were labeled using fluorescein isothiocyanate (FITC)-conjugated anti-rabbit secondary antibodies (Jackson, 1:1000). The cell nuclei were stained using 4′,6-diamidino-2-phenylindole (DAPI) (Beyotime, China).

#### Correlation between lncRNA expression and the INSS stage

Total RNA was extracted from the neuroblastoma tissue of 32 patients, and the expression of target lncRNAs was quantified by qRT-PCR. The INSS stage of each neuroblastoma tissue was confirmed by clinical diagnosis and therapy. Spearman correlation analyses were used to assess correlations between the expressions of lncRNAs of interest and the INSS stage.

#### Statistics

A t-test was used to analyze the results of the cell proliferation assays and gene and protein expression data. The χ^2^ test was used analyze the results of the apoptosis assays. Spearman correlation analyses were used to analyze the correlations between lncRNA expression levels and the INSS stage.

## Additional Information

**How to cite this article**: Tang, W. *et al*. MEG3, HCN3 and linc01105 influence the proliferation and apoptosis of neuroblastoma cells via the HIF-1α and p53 pathways. *Sci. Rep.* **6**, 36268; doi: 10.1038/srep36268 (2016).

**Publisher’s note:** Springer Nature remains neutral with regard to jurisdictional claims in published maps and institutional affiliations.

## Supplementary Material

Supplementary Information

## References

[b1] Park J. R., Eggert A. & Caron H. Neuroblastoma: biology, prognosis, and treatment. Hematol Oncol Clin NORTH Am. 24, 65–86 (2010). 20113896 10.1016/j.hoc.2009.11.011

[b2] Hogarty M. D. . Analysis of genomic imprinting at 1p35-36 in neurolastoma. Med Pediatr Oncol. Jan. 36(1), 52–55 (2001). 10.1002/1096-911X(20010101)36:1<52::AID-MPO1014>3.0.CO;2-8 11464906

[b3] Pajtler K. W. . Neuroblastoma in dialog with its stroma:NTRK1 is a regulator of cellular cross-talk with Schwann cells. Oncotarget 5(22), 11180–11192 (2014). 25361003 10.18632/oncotarget.2611 PMC4294349

[b4] Birney E. . Identification and analysis of functional elements in 1% of the human genome by the ENCODE pilot project. Nature 447, 799–816 (2007). 17571346 10.1038/nature05874 PMC2212820

[b5] Khachane A. N. & Harrison P. M. Mining mammalian transcript data for functional Long non-coding RNAs. PloS One 5(4), e10316 (2010). 20428234 10.1371/journal.pone.0010316 PMC2859052

[b6] Mercer T. R., Dinger M. E. & Mattick J. S. Long non-coding RNAs: insights into functions. Nat Rev Genet. 10, 155–159 (2009). 19188922 10.1038/nrg2521

[b7] Guangfu J. . Human polymorphisms at long non-coding RNAs(lncRNAs) and association with prostate cancer risk. Carcinogenesis. 32(11), 1655–1659 (2011). 21856995 10.1093/carcin/bgr187 PMC3204347

[b8] Zhou M. . Discovery of potential prognostic long non-coding RNA biomarkers for predicting the risk of tumor recurrence of breast cancer patients. Sci Rep. 6, 31038 (2016). 27503456 10.1038/srep31038 PMC4977495

[b9] Wang L. . Long non-coding RNA TUG1 promotes colorectal cancer matastasis via EMT pathway. Oncotarget. 32, 51713–51719 (2016). 10.18632/oncotarget.10563 PMC5239509 27421138

[b10] Xia H. . The lncRNA MALAT1 is a novel biomarker for gastric cancer metastasis. Oncotarget. 10, 18632 (2016). 10.18632/oncotarget.10941 PMC5302908 27486823

[b11] Øromua . Long noncoding RNAs with enhancer-like function in human cells. Cell. 143(1), 46–58 (2010). 20887892 10.1016/j.cell.2010.09.001 PMC4108080

[b12] Qureshi I. A., Mattick J. S. & Mehler M. F. Long non-coding RNAs in nervous system function and disease. Brain Res. 1338, 20–35 (2010). 20380817 10.1016/j.brainres.2010.03.110 PMC2883659

[b13] Sparago A. . Microdeletions in the human H19 DMR result in loss of IGF2 imprinting and Beckwith-Wiedemann syndrome. Nat Genet. 36, 958–960 (2004). 15314640 10.1038/ng1410

[b14] Johnson R. . Regulation of neural macroRNAs by the transcriptional repressor REST. RNA 15(1), 85–96 (2009). 19050060 10.1261/rna.1127009 PMC2612765

[b15] González W. & Bautista R. E. Seizures and EEG findings in an adult patient with DiGeorge syndrome: a case report and review of the literature. Seizure. 18(9), 648–651 (2009). 19665396 10.1016/j.seizure.2009.07.003

[b16] Willingham A. T. . A strategy for probing the function of noncoding RNAs finds a repressor of NFAT. Science 309(5740), 1570–1573 (2005). 16141075 10.1126/science.1115901

[b17] Triche T. J. Neuroblastoma–biology confronts nosology. Arch Pathol Lab Med. 110, 994–996 (1986). 3778134

[b18] Cheung N. & Dyer M. Neuroblastoma: developmental biology, cancer genomics and immunotherapy. Nat Rev Cancer. 13(6), 397–411 (2013). 23702928 10.1038/nrc3526 PMC4386662

[b19] Reinert L. S. . MCT-1 protein interacts with the cap complex and modulates messenger RNA translational profiles. Cancer Res. 66(18), 8994–9001 (2006). 16982740 10.1158/0008-5472.CAN-06-1999

[b20] Chen L. . lncRNA GAS5 is a critical regulator of metastasis phenotype of melanoma cells and inhibits tumor growth *in vivo*. OncoTarget and Therapy 9, 4075–4087 (2016). 10.2147/OTT.S98203 PMC4938146 27445498

[b21] Zhou Y., Wang D. L. & Pang Q. Long noncoding RNA SPRY4-IT1 is a prognostic factor for poor overall survival and has an oncogenic role in glioma. Eur Rev Med Pharmacol. Sci. 20(14), 3035–3039 (2016). 27460732

[b22] Gupta R. A. . Long non-coding RNA HOTAIR reprograms chromatin state to promote cancer metastasis. Nature 464, 1071–1076 (2010). 20393566 10.1038/nature08975 PMC3049919

[b23] Yu W. . Epigenetic silencing of tumour suppressor gene p15 by its antisense RNA. Nature. 451, 202–206 (2008). 18185590 10.1038/nature06468 PMC2743558

[b24] Ji P. . MALAT-1, a novel noncoding RNA, and thymosin beta 4 predict metastasis and survival in early-stage non-small cell lung cancer. Oncogene. 22, 8031–8041 (2003). 12970751 10.1038/sj.onc.1206928

[b25] Yamada K. . Phenotypic characterization of endometrial stromal sarcoma of the uterus. Cancer Sci. 97, 106–112 (2006). 16441420 10.1111/j.1349-7006.2006.00147.x PMC11158577

[b26] Lin R. . Alarge noncoding RNA is a marker for murine hepatocellular carcinomas and a spectrum of human carcinomas. Oncogene. 26, 851–858 (2007). 16878148 10.1038/sj.onc.1209846

[b27] Guffanti A. . A trascriptional sketch of a primary huan breast cancer by 454 deep sequencing. BMC Genomics 10, 163 (2009). 19379481 10.1186/1471-2164-10-163 PMC2678161

[b28] Wang J. . CREB up-regulates long non-coding RNA, HULC expression through interaction with microRNA-327 in liver cancer. Nucleic Acid Res. 38, 5366–5383 (2010). 20423907 10.1093/nar/gkq285 PMC2938198

[b29] DiFrancesco D. The contribution of the ‘pacemaker’ current (if) to generation of spontaneous activity in rabbit sino-atrial node myocytes. J Physiol. 434, 23–40 (1991). 2023118 10.1113/jphysiol.1991.sp018457 PMC1181405

[b30] Pape H. C. Queer current and pacemaker: the hyperpolarizationactivated cation current in neurons. Annu Rev Physiol. 58, 299–327 (1996). 8815797 10.1146/annurev.ph.58.030196.001503

[b31] Zhou Y., Zhang X. & Klibanski A. MEG3 noncoding RNA: a tumor suppressor. J. Mol. Endocrinol. 48, R45–R53 (2012). 22393162 10.1530/JME-12-0008 PMC3738193

[b32] Tian Z. Z. . Decreased expression of long non-coding RNA MEG3 acts as a potential predictor biomarker in progression and poor prognosis of asteosarcoma. Int J Clin Exp Pathol. 8(11), 15138–15142 (2015). 26823857 PMC4713643

[b33] Hammond E. M. & Giaccia A. J. The role of p53 in hypoxia-induced apoptosis. Biochem Biophys Res Commun. 331, 718–725 (2005). 15865928 10.1016/j.bbrc.2005.03.154

[b34] An W. G. . Stabilization of wild-type p53 by hypoxia-inducible factor 1alpha. Nature. 392, 405–408 (1998). 9537326 10.1038/32925

[b35] Carmeliet P. . Role of HIF-1alpha in hypoxia-mediated apoptosis, cell proliferation and tumour angiogenesis. Nature 394, 485–490 (1998). 9697772 10.1038/28867

[b36] Zhong H. . Overexpression of hypoxia-inducible factor 1 alpha in common human cancers and their metastases. Cancer Res. 59, 5830–5835 (1999). 10582706

[b37] Koong A. C. . Pancreatic tumors show high levels of hypoxia. Int J Radiat Oncol Biol Phys. 48, 919–922 (2000). 11072146 10.1016/s0360-3016(00)00803-8

[b38] Benetatos L., Vartholomatos G., Eleftheria H. & Hatzimichael E. MEG3 imprinted gene contribution in tumorigenesis. Int J Cancer 129(4), 773–779 (2011). 21400503 10.1002/ijc.26052

[b39] Fried H. U., Kaupp U. B. & Muller F. Benjamin Kaupp.Hyperpolarization-activated and cyclic nucleotide-gated channels are differentially expressed in juxtaglomerular cells in the olfactory bulb of mice. Cell Tissue Res. 339, 463–479 (2010). 20140458 10.1007/s00441-009-0904-9 PMC2838509

[b40] Wang W. . Expression and correlation of hypoxia-inducible factor-1alpha, vascular endothelial growth factor and microvessel density in experimental rat hepatocarcinogenesis. J Int Med Res. 37, 417–425 (2009). 19383236 10.1177/147323000903700217

[b41] Sasabe E., Tatemoto Y., Yamanoto T. & Osaki T. Mechanism of HIF-1alpha-dependent suppression of hypoxia-induced apoptosis in squamous cell carcinoma cells. Cancer Sci. 96, 394–402 (2005). 16053510 10.1111/j.1349-7006.2005.00065.x PMC11158431

[b42] Youle R. J. & Strasser A. The BCL-2 protein family: opposing activities that mediate cell death. Nat Rev Mol Cell Biol. 9, 47–59 (2008). 18097445 10.1038/nrm2308

[b43] Chen L. . Differential targeting of prosurvival Bcl-2 proteins by their BH3-only ligands allows complementary apoptotic function. Mol Cell. 7, 393–403 (2005). 10.1016/j.molcel.2004.12.030 15694340

[b44] Bonzon C., Hayes L. B., Pagliari L. D. & Newmeyer D. Caspase-2-induced apoptosis requires bid cleavage: a physiological role for bid in heat shock-induced death. Mol. Biol. Cell. 17, 2150–2157 (2006). 16495337 10.1091/mbc.E05-12-1107 PMC1446087

[b45] Li H., Zhu H., Xu C. J. & Yuan J. Cleavage of BID by caspase 8 mediates the mitochondrial damage in the Fas pathway of apoptosis. Cell 94, 491–501 (1998). 9727492 10.1016/s0092-8674(00)81590-1

[b46] Luo X. . Bid, a Bcl2 interacting protein, mediates cytochrome c release from mitochondria in response to activation of cell surface death receptors. Cell. 94, 481–490 (1998). 9727491 10.1016/s0092-8674(00)81589-5

[b47] Maas C., Vries E. D., Tait S. W. G. & Borst J. Bid can mediate a pro-apoptotic response to etoposide and ionizing radiation without cleavage in its unstructured loop and in the absence of p53. Oncogene 30, 3636–3647 (2011). 21423217 10.1038/onc.2011.75 PMC3158540

[b48] Plesnila N. . BID mediates neuronal cell death after oxygen/glucose deprivation and focal cerebral ischemia. Proc. Natl. Acad. Sci. USA 98, 15318–15323 (2001). 11742085 10.1073/pnas.261323298 PMC65027

[b49] Upton J. P. . Caspase-2 cleavage of BID is a critical apoptotic signal downstream of endoplasmic reticulum stress. Mol. Cell. Biol. 28, 3943–3951 (2008). 18426910 10.1128/MCB.00013-08 PMC2423129

[b50] Lu K. H. . Long non-coding RNA MEG3 inhibits NSCLC cells proliferation and induces apoptosis by affecting p53 expression. BMC Cancer 13, 461 (2013). 24098911 10.1186/1471-2407-13-461 PMC3851462

