# Supplementary material for: MEG3, HCN3 and linc01105 influence the proliferation and apoptosis of neuroblastoma cells via the HIF-1α and p53 pathways
Source: Sci Rep. 2016 Nov 8;6:36268. doi: 10.1038/srep36268 (PMC5099956; doi:10.1038/srep36268)
Supplement: Supplementary Information [file srep36268-s1.pdf]

MEG3, HCN3 and linc01105 influence the proliferation and apoptosis of neuroblastoma cells via the HIF-1α and p53 pathway

Weitao Tang, Kuiran Dong, Kai Li, Rui Dong, Shan Zheng

Supplementary figure 1. Heat map and hierarchical cluster analysis of neuroblastoma and para-tumor.

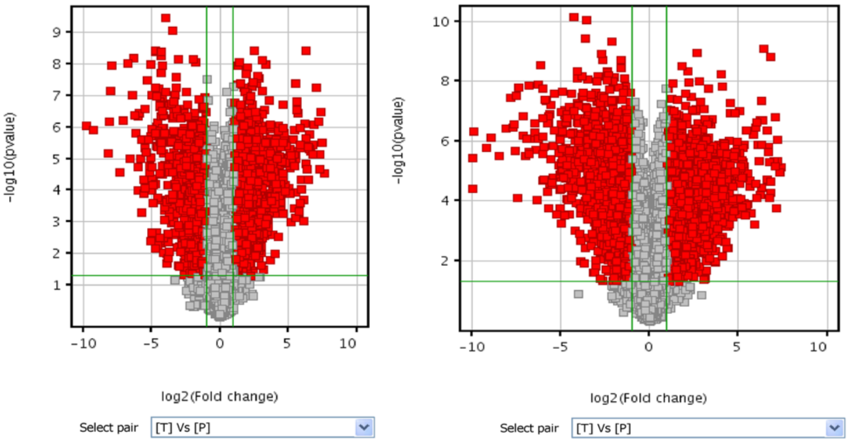

Supplementary figure 1: Heat map representing expression values of a panel of neuroblastoma group to para-tumor group. Each column represents the indicated one sample. Each row indicates mRNAs(right) or lncRNAs(left). The color change reflects relative change according to the scale shown; red indicates positive fold change and green indicates negative fold change.

Supplementary figure2. Gene expression in neroblastoma and para-tumor.

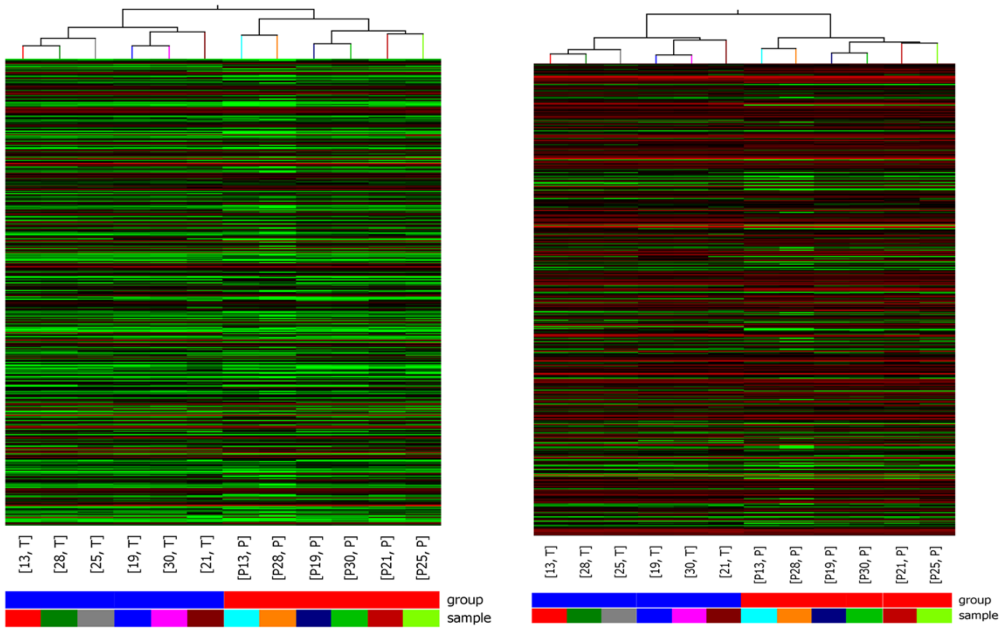

Supplementary figure2: Volcano Plots are useful tools for visualizing differential expression between two different conditions. They are constructed using fold-change values and P-values, and thus allow you to visulaize the relationship between fold-change (magnitude of change) and statistical significance (which takes both magnitude of change and variability into consideration).

They also allow subsets of genes to be isolated, based on those values. The vertical lines correspond to 2.0-fold up and down and the horizontal line represents a P-value of 0.05. So the red point in the plot represents the differentially expressed lncRNAs (left) or mRNA(right) with statistical significance.

Supplementary figure3. CNC network analysis

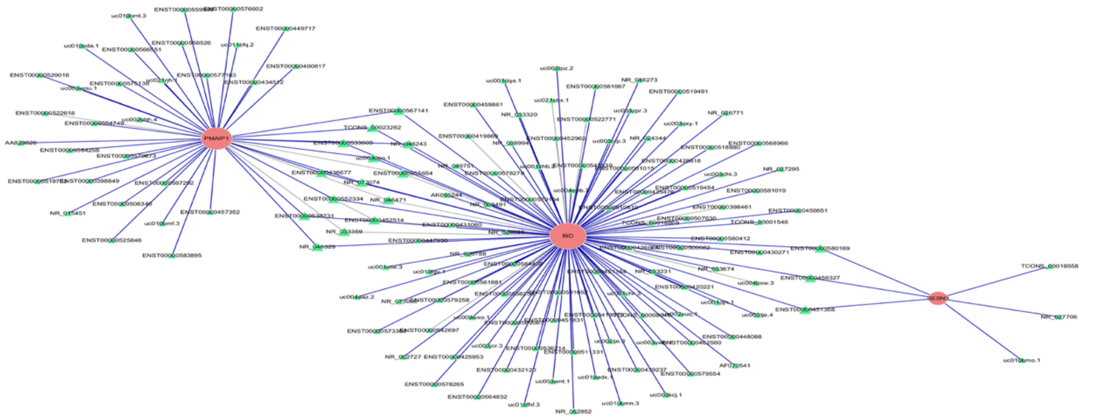

Supplementary figure3: The red code represents mRNAs, and those that collected with red code represents lncRNAs. The blue lines represent positive correlation and the gray lines represent negative correlation.

Supplementary figure4. Confirmation of three differentially expressed lncRNAs by Real-Time PCR

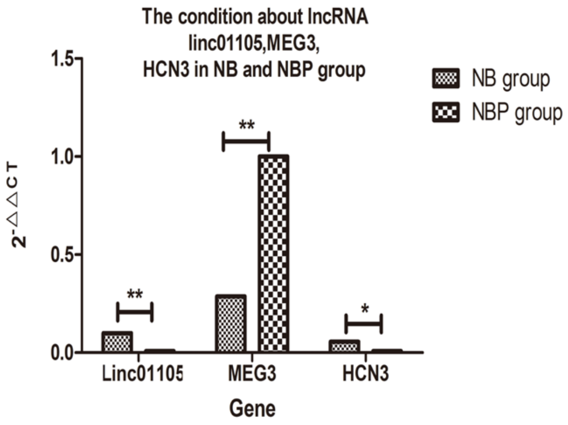

Supplementary figure4: In the nueroblastoma tissues, the level of the lncRNA-HCN3 and linc01105 was higher than that in the para-tumor tissues (P＜0.01).

Supplementary figure 5. Multiple exposures of blots

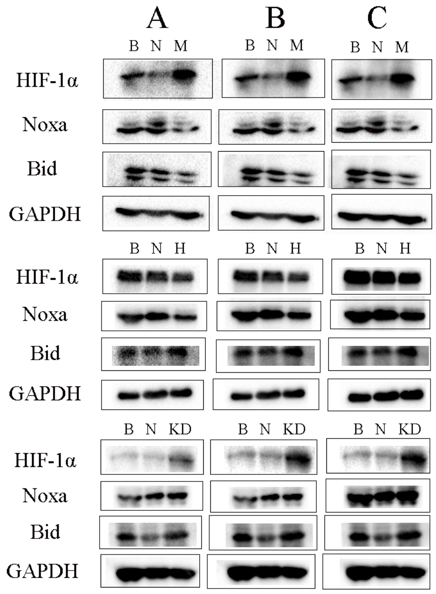

Supplementary figure5: Line A, B and C represent multiple exposures of blots. B, Blank group; N, Negative control group; M, MEG3 OE group; H, HCN3 KD group; KD, linc01105 KD group.

Supplementary figure 6. The figures of full length blots.

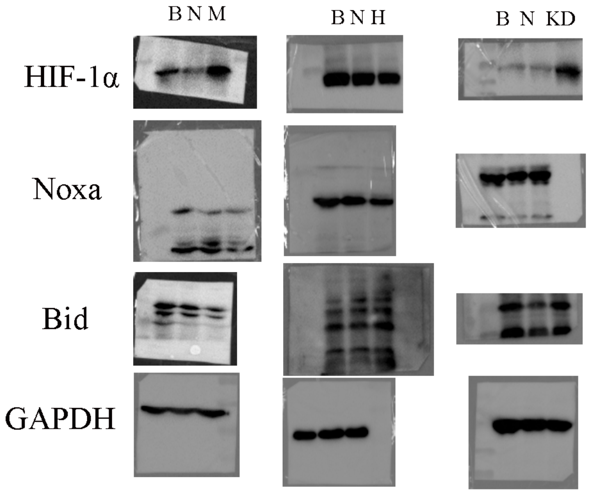

Supplementary figure6: B, Blank group; N, Negative control group; M, MEG3 OE group; H, HCN3 KD group; KD, linc01105 KD group.
